# New insights on the complex dynamics of two-phase flow in porous media under intermediate-wet conditions

**DOI:** 10.1038/s41598-017-04545-4

**Published:** 2017-07-04

**Authors:** Harris Sajjad Rabbani, Vahid Joekar-Niasar, Tannaz Pak, Nima Shokri

**Affiliations:** 1 School of Chemical Engineering and Analytical Science, The University of Manchester, Manchester, M13 9PL United Kingdom; 2 School of Science & Engineering, Teesside University, Middlesbrough, TS1 3BX United Kingdom

## Abstract

Multiphase flow in porous media is important in a number of environmental and industrial applications such as soil remediation, CO_2_ sequestration, and enhanced oil recovery. Wetting properties control flow of immiscible fluids in porous media and fluids distribution in the pore space. In contrast to the strong and weak wet conditions, pore-scale physics of immiscible displacement under intermediate-wet conditions is less understood. This study reports the results of a series of two-dimensional high-resolution direct numerical simulations with the aim of understanding the pore-scale dynamics of two-phase immiscible fluid flow under intermediate-wet conditions. Our results show that for intermediate-wet porous media, pore geometry has a strong influence on interface dynamics, leading to co-existence of concave and convex interfaces. Intermediate wettability leads to various interfacial movements which are not identified under imbibition or drainage conditions. These pore-scale events significantly influence macro-scale flow behaviour causing the counter-intuitive decline in recovery of the defending fluid from weak imbibition to intermediate-wet conditions.

## Introduction

The physics of immiscible two-phase flow in porous media is a subject of intense study in a number of applications including enhanced oil recovery^1^, CO_2_ sequestration^2^, remediation of contaminated aquifers^3^, drying of porous media^4^ and drug delivery^5^. Wettability, defined as the tendency of a fluid to spread over a solid surface in the presence of another fluid^6^, has a significant impact on the dynamics of immiscible displacement^7–10^. The wetting conditions of a solid surface – as a result of the relative importance of the adhesive and cohesive forces - can be classified into strong-wet, intermediate-wet, and weak-wet. In strong-wet and weak-wet cases, one of the fluids has substantial preferential affinity to a solid surface. Alternatively, if both fluids have similar affinity to the solid surface, the surface is referred to as intermediate-wet. Under strong and weak wet conditions, fluid displacement processes in porous media are referred as drainage and imbibition. In drainage, the defending fluid is the wetting phase, while in imbibition the invading fluid wets the solid surface. The pore-scale displacement mechanisms that have been identified to occur in strong and weak wet porous media are snap-off^11, 12^, piston like displacement^13^, corner flow^8^, cooperative pore filling^7^, Haines jump^14^ and droplet fragmentation^15^. However, pore-scale displacement for intermediate-wet conditions remains less understood, despite the fact that intermediate-wet conditions occur in many natural porous media^9^.

Here, we use Computational Fluid Dynamics (CFD) modelling to perform direct numerical simulation of two-phase immiscible fluids displacement in a porous medium, which is designed based on the pore-scale X-ray tomography image of a real sand pack. Performing direct numerical simulations on 2D^16, 17^ and 3D^18, 19^ images of real porous media is an advanced tool that allows capturing more detailed fluid dynamics information compared to pore-network modelling approach^20–23^, specifically for complex pore morphologies. We present results of direct 2D numerical simulations performed on a wide range of wettability conditions with a particular focus on intermediate-wet condition. Our results demonstrate the co-existence of concave and convex interfaces under intermediate-wet conditions emanated from the interplay between the wetting characteristics and pore geometry. Such a phenomenon promotes (i) pinning of convex interface, (ii) pore-level reverse displacement and (iii) interface instability. These complex yet intriguing pore-scale displacement events provide novel explanations to the classical non-monotonic behaviour of recovery of defending fluid as a function of porous media wettability.

## Direct numerical simulations

Immiscible two-phase flow is simulated through a heterogeneous 2D porous medium (patterns presented in Supplementary Information, Fig. S1). The Navier-Stokes equation coupled with Volume of Fluid algorithm (interface tracking approach) is numerically solved using OpenFoam (Open Field Operation and Manipulation). Complete information on the equations governing multiphase flow in porous media is provided in Supplementary section. The wettability of porous media is defined by the contact angle, θ, between the fluid-fluid interface (through the invading phase) and the grain surface, which is an input parameter to the solver. A series of numerical simulations are performed with different θ values ranging from 5° to 140°. Contact angle ranging from 5° to 15° represents strong-imbibition, from 30° to 45° indicates weak-imbibition, from 60° to 100° shows intermediate-wet and from 120° to 140° represents drainage condition. In order to eliminate the effect of contact angle hysteresis, the advancing and receding θ are kept equal, resulting in uniform distribution of contact angle across the simulation domain. The invading fluid (with the viscosity of 0.001 Pa.s) is injected in the porous medium initially saturated with defending fluid (with the viscosity of 0.008 Pa s) at a constant flow rate of 1.8 ml/hr for 6.5 s.

## Results and Discussion

### Intermediate-wet porous media and interface dynamics

**Figure 1:**
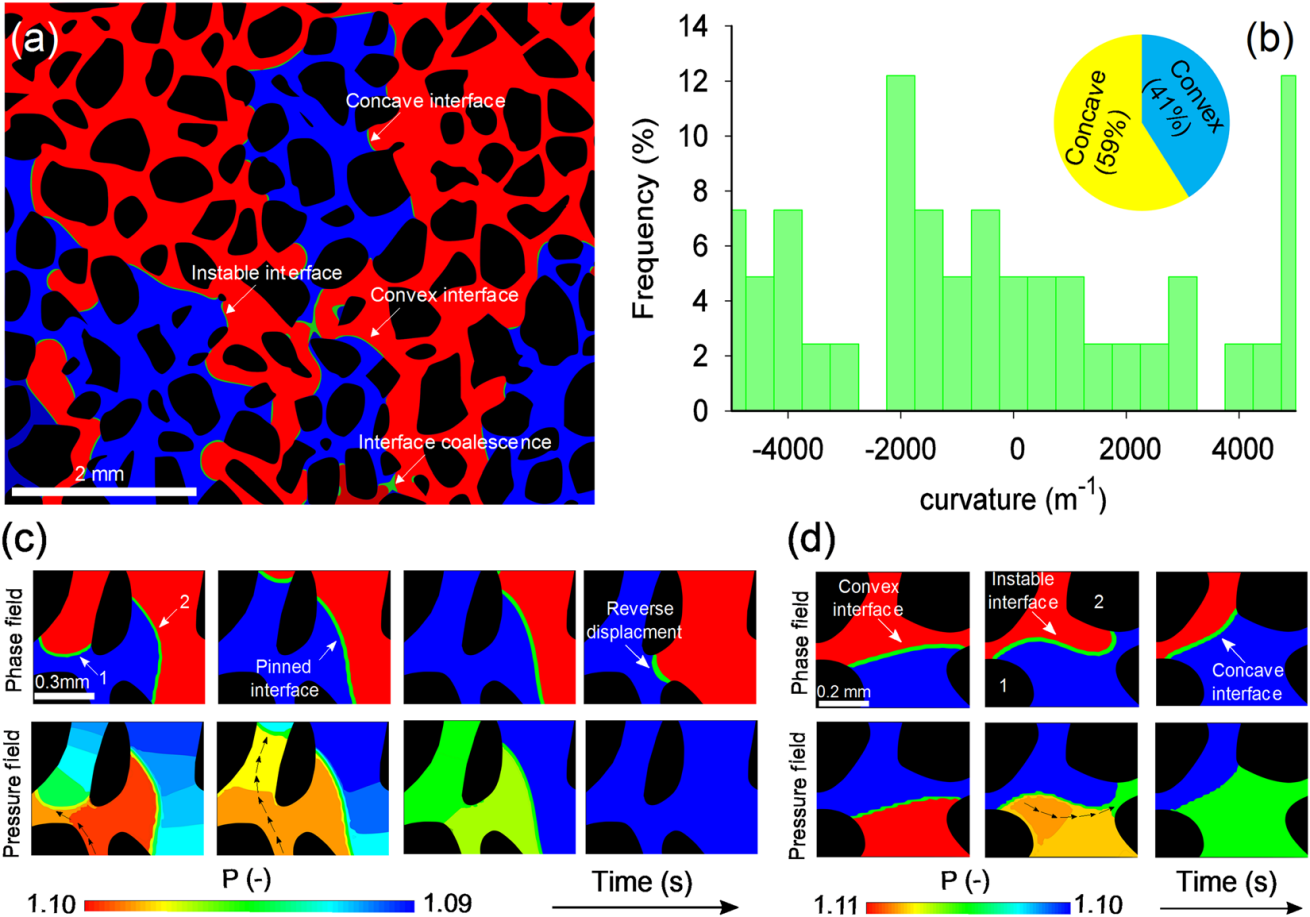
(**a**) The main interfacial features observed during immiscible two-phase flow in intermediate-wet porous media (θ = 60°) at 2.8 s. (**b**) Curvature distribution of interfaces shown in Fig. 1(a). (**c**) Dynamics of concave (labelled as “1”) and convex (labelled as “2”) interfaces during displacement in the porous medium with θ = 60°. Pinning of convex interface and reverse displacement mechanism as a result of co-existence of concave and convex interface is observed. (**d**) Interface instability in a single pore. In the phase distribution shown in Fig. 1(a,c,d), red, blue and green represents defending fluid, invading fluid and the fluid-fluid interface, respectively. The pressure field shown in Fig. 1(c–d) indicates the pressure values normalized with respect to the outlet pressure. The direction of injection in all images is from bottom to top.

The capillary forces in intermediate-wet porous media are weak. This leads to occurrence of various interfacial phenomena that are not present in strong and weak wet porous media. The key interfacial feature observed under uniformly distributed contact angle in the range of 60°–100° is the presence of both concave and convex interfaces. This is illustrated in Fig. 1(a).

The co-existence of both concave and convex interfaces stems from the increasing dependence of the interface morphology on the angularity of pores (the angle at which a pore converges or diverges) as the wettability changes from strong-wet to intermediate-wet conditions. In other words, the complex interplay between contact angle and pore angularity influence the direction of capillary forces leading to the variations of the interface curvature^24–26^. Statistical analysis of interface curvature presented in Fig. 1(b) illustrates the comparison between the positive (convex) and negative (concave) curvature under intermediate-wet conditions.

The co-existence of concave and convex interfaces influences the displacement dynamics and overall flow pattern at micro and macro-scale. The micro-scale interface topology has been illustrated in Fig. 1(c,d). Figure 1(c) shows that while the concave interface (interface 1) is displaced upwards, the convex interface (interface 2) is temporarily “pinned” at the junction of pore body. The behaviour of the interfaces 1 and 2 can be explained using the computed pressure fields presented in Fig. 1(c). Due to the contrast in the morphology of the interfaces 1 and 2, the pressure gradient developed within the invading phase causes the preferential flow of invading phase towards interface 1. This restricts the displacement of interface 2 as shown in Fig. 1(c). Over time, pressure gradient across interface 2 declines; as a consequence, the interface is forced back into the pore throat against the direction of the main stream flow. Such a mechanism has been observed in Berg *et al*.^14^ and Joekar-Niasar *et al*.^27^. We refer to this phenomenon as the “reverse displacement”. It is important to note that as interface 2 enters the pore throat, its curvature changes from convex to concave. This analysis demonstrates the impact of pore angularity in dictating the curvature of the interface for the contact angle θ of 60°.

The obtained high resolution numerical results allow us to investigate another complex interfacial process occurring in intermediate-wet porous media that is related to the instability of interface in a single pore (Fig. 1(d)). As explained before, in the presence of intermediate-wet condition, the curvature of an interface can change from convex to concave or vice versa. Figure 1(d) illustrates that such morphological transformation of interface is not spontaneous, but occurs through an intermediate stage where the interface is instable. The morphology of the instable interface is significantly different from its stable counter parts that are concave and convex. Figure 1(d) shows that across one single interface, the sign of capillary pressure (defined by the difference between the pressures across the interface) changes. At macroscopic-scale, this will lead to non-uniform distribution of the capillary pressure.

The instable interface depicted in Fig. 1(d) manifests that near the pore wall 1, the interface is convex, while at the pore wall 2 the interface is concave. The sharp variation in the curvature of interface induces pressure gradient within invading phase similar to what has been discussed before. However, unlike the case illustrated in Fig. 1(c), both concave and convex sides of the interface are attached and facilitates the movement of each other exhibiting cooperative behaviour. As a result of the pressure gradient, the invading phase tends to flow from high pressure region (convex) to low pressure region (concave) indicated with black arrows in Fig. 1(d). This ceases the advancement of convex interface momentarily, but provides impetus for the concave interface to move forward.

### Non-monotonic recovery of defending fluid as a function of wettability

**Figure 2:**
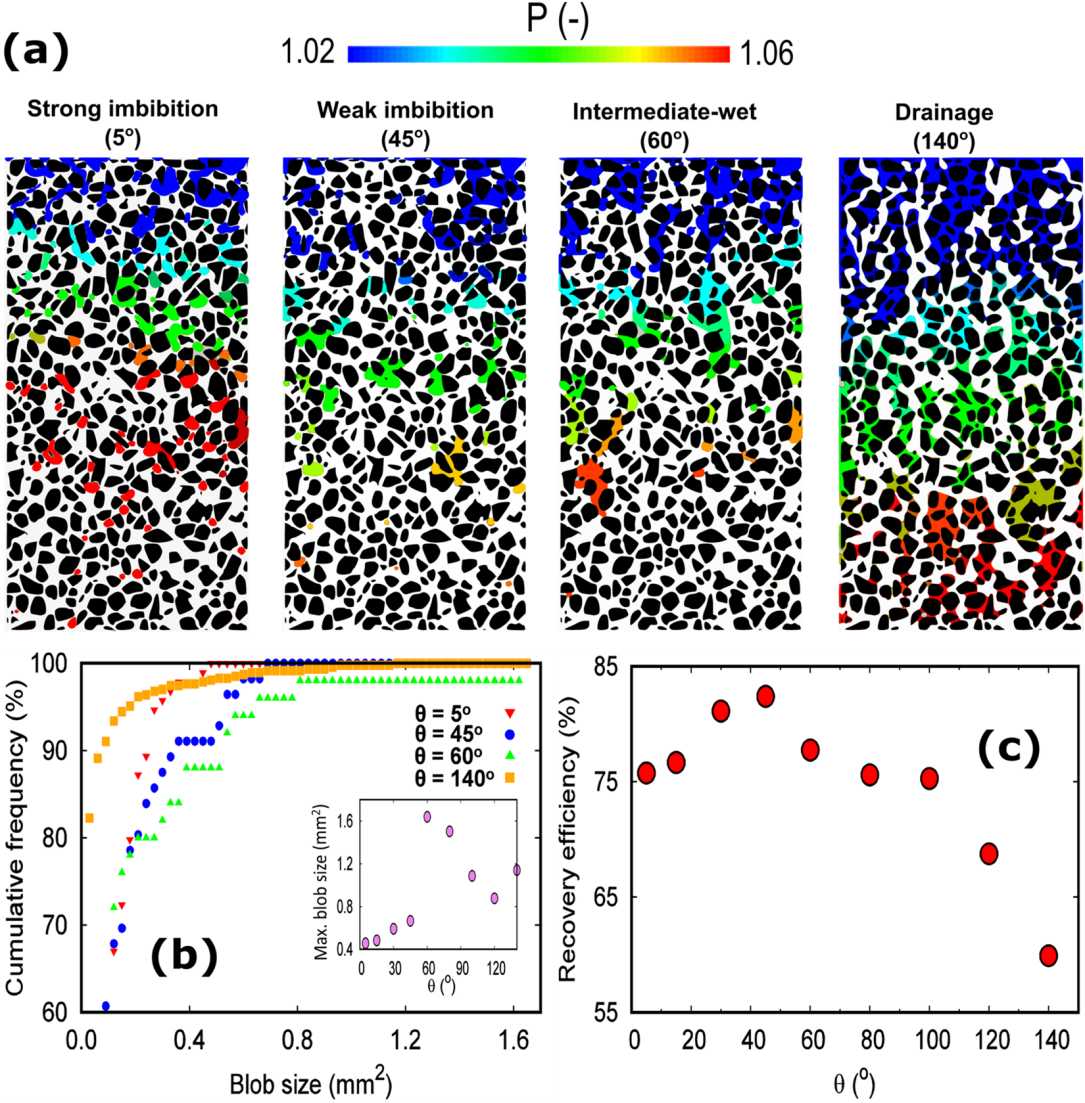
(**a**) Fluid phase and pressure distribution under different wetting conditions at the end of simulation. White colour represents pathway of invading phase. Pressure is normalized with respect to the outlet pressure and it indicates the pressure in the defending phase. (**b**) Distribution of blobs size of defending fluid under different wettability scenarios. The inset illustrates the maximum blob size as a function of the contact angle. (**c**) The non-monotonic dependency of the defending phase recovery on the wettability of porous media.

Under intermediate-wet conditions, interaction of interface with pore surface leads to the co-existence of concave and convex interface (Fig. 1(a)) which has been observed in different pores (Fig. 1(c)) and even within a single irregular pore (Fig. 1(d)). To investigate the influence of these displacement events on the macroscopic flow behaviour, we quantified the recovery efficiency of the defending fluid as a function of the wettability of porous media with the results being presented in Fig. 2.

Figure 2(a) shows the distribution of phases under different wetting conditions. Visual inspection of this figure along with Fig. 2(b) shows that under intermediate-wet conditions the blobs of defending fluid are more widespread compared to other wetting conditions which might be attributed to the interface coalescence. Furthermore, the recovery efficiency of the defending fluid (the area represented by white in Fig. 2(a)) as a function of the wettability of porous media is quantified and shown in Fig. 2(c).

Traditionally, the contact angle measured on flat surface is known to be a major indicator of change in wettability of porous media^8^, which can be mathematically defined according to Young-Dupre law, i.e. *σ*_1_ = *σ*_*o*_ cos(θ) + σ_2_ where *σ*_1_ is the surface tension between defending fluid and solid surface, *σ*_*o*_ is the interfacial tension between invading and defending fluid and *σ*_2_ is the surface tension between invading fluid and solid surface. Since on the basis of Young-Dupre law, the capillary forces are the weakest under intermediate-wet conditions (or to be more specific at contact angle θ of 90°), one may expect the highest recovery efficiency under intermediate-wet condition. However, our results do not support this conclusion. As indicated in Fig. 2(c), the recovery efficiency is a non-monotonic function of wettability of porous media, but the highest recovery efficiency is found to be under weak imbibition conditions. Figure 2(c) shows that the recovery of defending fluid reduces when the contact angle θ increases from 45° to 100° which is counter intuitive. Although, the trend indicated in Fig. 2(c) has been observed previously by Ryazanov *et al*.^28^ and Zhao *et al*.^8^, the underlying physical processes were remained elusive which are discussed next.

Our numerical results delineate the underlying mechanisms of the counter-intuitive decline of defending-fluid recovery from the weak imbibition to the intermediate-wet condition. We found that this non-monotonic behaviour is governed by a critical contact angle θ_c_. The critical contact angle when the arc interface (i.e. the interface residing in corners of pores) is flat is a function of the corner angle^29^. The relationship between corner angle of pore and critical contact angle θ_c_ can be mathematically defined as ${{\rm{\theta }}}_{{\rm{c}}}={\rm{\pi }}-\frac{\beta }{2}$, where β is the corner angle. For a typical micro-model (which is the simulation domain of present investigation), β = 90° which results in θ_c_ = 45°. Detailed analysis of the role of corner angle on capillary pressure and interface dynamics under various wetting conditions has been presented in Ma *et al*.^29^ and Rabbani *et al*.^30^ thus not repeated here. According to Fig. 2(c), the maximum recovery in our system occurs at weak imbibition condition (at the contact angle close to 45°) which is indeed in agreement with the microfluidic experimental results reported in Zhao *et al*.^8^ and 3D investigation performed by Singh *et al*.^31^. Furthermore, Fig. 1 suggests that under intermediate-wet conditions (θ = 60°–100°), pore angularity (i.e. converging-diverging angle) plays a crucial role in dictating the curvature of the interface. Different direction of capillary forces acting along the interfaces that are residing in different pores induces dramatic decline in the mobility of convex interface which eventually reduces the recovery efficiency (Fig. 1(c)). Although, interface instability shown in Fig. 1(d) can be regarded as a phenomenon that inhibits the entrapment of defending phase (due to cooperative behaviour of concave and convex interface), its influence is localized within single pores. In contrary to the interface instability, the effects of pinning of convex interfaces and reverse displacement phenomena (as a consequence of pinned convex interface) shown in Fig. 1(c) dominate the dynamics of displacement in intermediate-wet condition.

Since the conventional Young-Dupre law does not accommodate the role of pore geometry (corner angle and converging-diverging angle), the characterization of recovery efficiency curves by mere definition of wettability based on the flat surfaces can be misleading and can obscure the true physics controlling the recovery curve (as illustrated in our results obtained by the direct numerical simulation).

## Summary and Conclusions

Wetting characteristics of porous media significantly influence multiphase flow and transport processes. In the present study, we conducted a comprehensive series of investigation by means of direct numerical simulation to delineate the pore-scale mechanisms controlling immiscible two-phase flow in porous media under different wettability scenarios with a particular focus on intermediate-wet conditions which has been rarely discussed in literature. The present pore-scale analysis helps to rationalize the physics governing some of the unexplained previous observations^8, 28^. With the current experimental tools available, it is not feasible to experimentally observe some of the effects induced by the wettability condition which ultimately determine the dynamics of displacement in porous media (such as the pressure field developed at pore-scale influencing the interface dynamics as illustrated in Fig. 1(c,d)). Inspection and visualization of our numerical results enabled us to gain insights on the complex pore level dynamics controlling the displacement mechanisms as a function of wetting properties of porous media and the resulting macroscopic displacement patterns that emerge.

Our numerical results revealed a non-monotonic dependence of defending fluid recovery on the wetting characteristics of porous media with the recovery efficiency being the highest under the weak imbibition condition. At pore-scale, our results confirms the presence of both concave and convex interfaces under intermediate-wet conditions. We show that for a uniform contact angle, both concave and convex interface exists in heterogeneous porous media. This co-existence of concave and convex interface leads to several interfacial processes influencing the dynamics of multiphase flow. The illustrated processes including pinning of convex interface and reverse displacement causes decline in the recovery efficiency of defending fluid.

Furthermore, we illustrate that linking the contact angle measured on flat surfaces to the recovery efficiency of defending fluid is not sufficient to describe the governing mechanisms and that the geometry of pore is another important parameter which must be taken into consideration that controls the recovery efficiency.

## Materials and Methods

### Simulation domain

We have used pore-scale images obtained by 3D X-ray micro-tomography of a real sand pack^32^ as the simulation domain. The 2D image that was used for simulation in the present study is shown in Fig. S1 of the Supplementary information which illustrates the grain arrangement at the central cross section of the sand pack. More information about the pore and grain size is given in Table S1 of the Supplementary information. Rhinoceros (CAD software) was used to extract the pore network skeleton from digital images of the porous medium which was imported into the simulator as an STL (STereoLithography) file.

The numerical domain was first converted into triangulated surface geometry, which was later discretised into small elements by means of the mesh generator in OpenFoam^16^. The final arrangement of these elements was almost unstructured, near the grain surface it was split-hexahedrals and hexahedrals elsewhere^16^. The meshing algorithm employed in this research has been successfully used by Ferrari *et al*.^33^. According to the grid independence analysis performed in Rabbani *et al*.^30^, the optimum size of the spatial element chosen for the computational domains scaled with respect to the average pore size was 0.1.

### Validation of the numerical simulation

**Figure 3:**
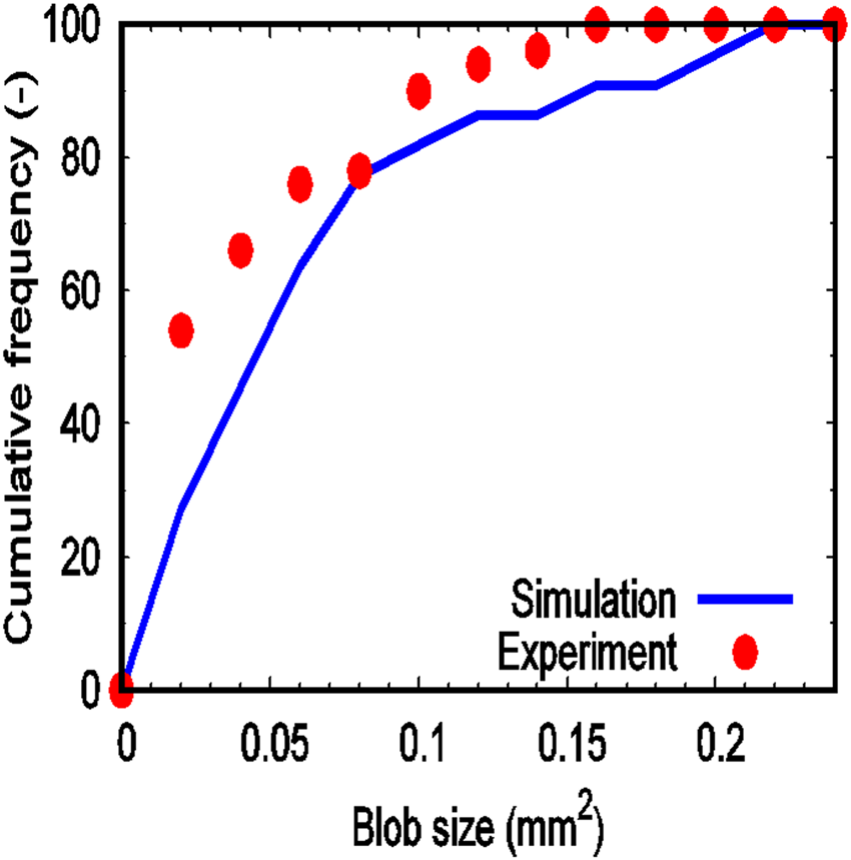
Comparison between the blob-size distributions computed numerically and the ones measured by the microfluidic experiments for the fluids PMX – 200 Silicone Fluid and water with water injection rate of 1.0 ml/hr.

In addition to the numerical simulations, microfluidics experiments were conducted to evaluate the performance of the numerical model. A micromodel was fabricated using the same pore-scale 2D image obtained by 3D X-Ray micro-tomography of a sand pack. The micromodel was fabricated in a silicon wafer using standard photolithography and inductively coupled plasma-deep reactive ion etching (ICP-DRIE) methods. Further detail about the fabrication procedure can be found in Willingham *et al*.^34^. The contact angle of the micromodel was 15.8°. The micromodel was saturated with PMX – 200 Silicone Fluid having viscosity of 1 × 10^−1^ Pa s (provided by Dow Corning) at flow rate of 100 ml/hr and then displaced by de-ionized water at 1.0 ml/hr. Dynamics of the displacement was recorded using an optical microscope (Leica M205C, 20.5:1 zoom, 0.925 µm resolution, equipped with a Leica DFC 3000G high resolution digital camera). More detail about the experimental procedure can be found in Rodríguez de Castro *et al*.^35, 36^. We have quantified the distribution of the trapped blobs of the defending fluid obtained by the simulation and experiment (results presented in Fig. 3).

The comparison shows that the numerical prediction slightly underestimates the experimental results. A possible explanation for this discrepancy could be related to the edges of the grains. In the micromodel, the grains could have some roughness, which is not presented in the domain used for the numerical simulation. Roughness of the grains enhances the entrapment of smaller blobs. Other possible reasons of this discrepancy could be related to the measured contact angle of the micromodel as well as the experimental values obtained based on the segmented images. These could be possible sources of the difference observed between numerically determined residual saturation (30.4%) and the experimentally measured value (32%).

## Electronic supplementary material

SUPPLEMENTARY INFORMATION
