# Supplementary material for: New insights on the complex dynamics of two-phase flow in porous media under intermediate-wet conditions
Source: Sci Rep. 2017 Jul 4;7:4584. doi: 10.1038/s41598-017-04545-4 (PMC5496885; doi:10.1038/s41598-017-04545-4)
Supplement: Supplementary file 1 — SUPPLEMENTARY INFORMATION [file 41598_2017_4545_MOESM1_ESM.pdf]

New insights on the complex dynamics of two-phase flow in porous media under intermediate-wet conditions

Harris Sajjad Rabbani[1],Vahid Joekar-Niasar[1], Tannaz Pak[2], Nima Shokri[1*]

[1]School of Chemical Engineering and Analytical Science, The University of Manchester, Manchester,M13 9PL, United Kingdom

[2]School of Science & Engineering, Teesside University, Middlesbrough, TS1 3BX, United Kingdom

Supplementary information

The porous medium used for numerical modelling in the present study is shown in Fig. S1.

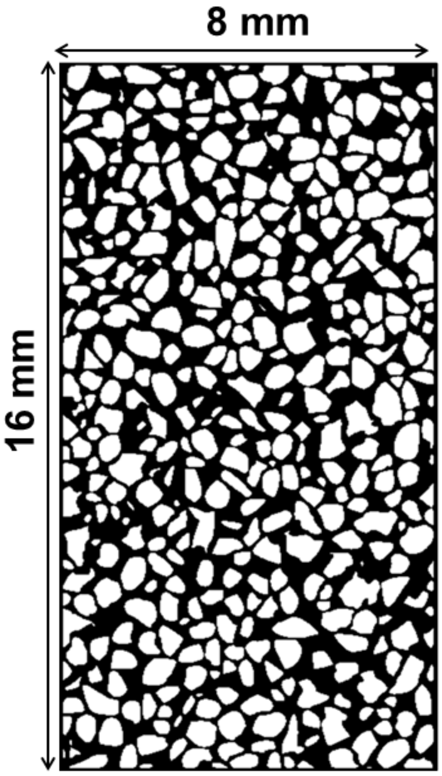

Figure S1. The porous medium (numerical domain) used for simulation of immiscible displacement under different wettability conditions. Black and white colours represent void and solid phase, respectively. The porous medium is based on the pore-scale image obtained by X-ray tomography of a sand pack[32].

In this research direct numerical simulation was performed using C++ library called OpenFoam (Open Field Operation and Manipulation).The pore scale dynamics of immiscible displacement is governed by mass and momentum equation, as follows[30];

Mass balance

$$\nabla . \mathbf{u} = 0 \tag{1.1}$$

Momentum balance

$$\frac{\partial \rho \mathbf{u}}{\partial t} + \nabla \cdot (\rho \mathbf{u}\mathbf{u}) = -\nabla p + \nabla \cdot (\mu(\nabla \mathbf{u} + \nabla \mathbf{u}^T) + f_{sa} \quad (1.2)$$

where **u** is the velocity vector (m s^{-1}), ρ is the density (kg m^{-3}), p is the pressure (kg m^{-1} s^{-2}), μ is the viscosity (kg m^{-1} s^{-1}) and f_{sa} is the capillary force (kg m^{-2} s^{-2}) acting as a source term.

Volume of fluid (VOF) was employed as an interface capturing approach. It defines the distribution of phases using volume indicator function γ expressed as;

$$\gamma \in \begin{cases} (0,1) & \text{Interface;} \\ (1) & \text{Defending fluid;} \\ (0) & \text{Invading fluid;} \end{cases}$$

The volume indicator function evolves according to advection transport as shown in Eq. (1.3):

$$\frac{\partial \gamma}{\partial t} + \nabla \cdot (\gamma u) + \nabla \cdot (\gamma(1-\gamma)u_r) = 0 \quad (1.3)$$

where u_r is the relative velocity between two fluids (m s^{-1}), also known as "compression velocity". In each computational cell, ρ and μ is a linear function of γ and varies according to phase present.

$$\rho = \gamma \rho_i + (1-\gamma)\rho_d \quad (1.4)$$

$$\mu = \gamma \mu_i + (1-\gamma)\mu_d \quad (1.5)$$

In Eq. 1.4 and 1.5, ρ_i is the density of invading phase (kg m^{-3}), ρ_d is the density of defending phase (kg m^{-3}), μ_i refers to viscosity of invading phase (kg m^{-1} s^{-1}) and μ_d is the viscosity of defending phase (kg m^{-1} s^{-1}). The term $f_{sa}$was described using Continuum Surface Force (CSF) method[30] as follows;

$$f_{sa} = \sigma_{12}\kappa\nabla\gamma\,\delta_s \quad (1.6)$$

where δ_s is the Dirac Function and κ is the curvature of the interface (m^{-1}) computed as;

$$\kappa = \nabla \cdot \left(\frac{\nabla\gamma}{|\nabla\gamma|}\right) \quad (1.7)$$

f_{sa} is only active at the interface and vanishes at limits of γ. All equations are discretized at each computational cell using finite volume method and solved numerically to obtain variables p, u and γ.

The post-processing of numerical results was conducted with Paraview; an open-source visualization application. For curvature calculation shown in Fig. 1, the contour map of interface with γ=0.5 is extracted. Then the built-in Paraview plugin was used to compute distribution of curvature at each interface. Finally, the curvature of whole interface was represented by the median value.

Table S1. Physical description of the porous medium as given in Norouzi Rad et al.[32]

Grain size range (mm)	Average grain size (mm)	Average pore size (mm)	Porosity (-)
0.36-0.77	0.60	0.16	0.37
